# Supplementary material for: Hybridization Capture Using RAD Probes (hyRAD), a New Tool for Performing Genomic Analyses on Collection Specimens
Source: PLoS One. 2016 Mar 21;11(3):e0151651. doi: 10.1371/journal.pone.0151651 (PMC4801390; doi:10.1371/journal.pone.0151651)
Supplement: S1 Fig — Left panel, X-axis: fragment size (semi-log scale); Y-axis: fragment density (Relative Fluorescent Units). Right panel, gel-like representation of the left panel. (DOCX) [file pone.0151651.s001.docx]

# Supporting information


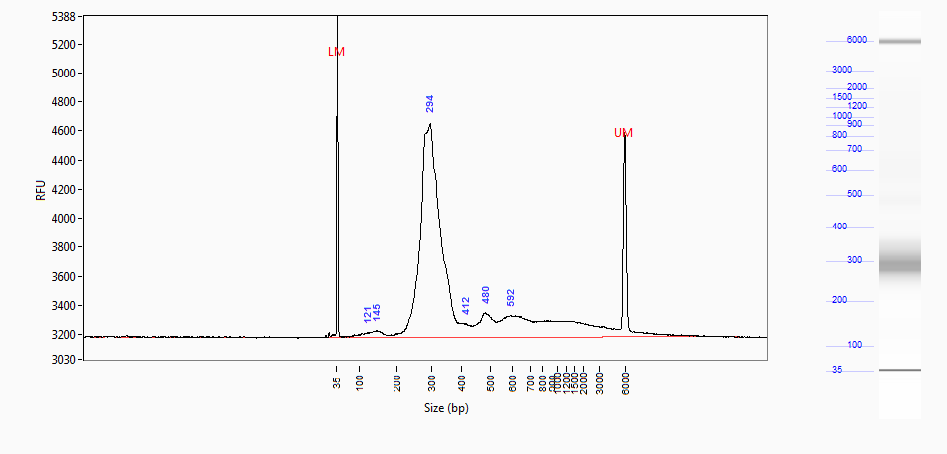


S1 Fig. Profile of the RAD-probes precursor, the RAD-seq library. Left panel, *X*-axis: fragment size (semi-*log* scale); *Y*-axis: fragment density (Relative Fluorescent Units). Right panel : gel-like representation of the left panel.
