# Supplementary material for: Hybridization Capture Using RAD Probes (hyRAD), a New Tool for Performing Genomic Analyses on Collection Specimens
Source: PLoS One. 2016 Mar 21;11(3):e0151651. doi: 10.1371/journal.pone.0151651 (PMC4801390; doi:10.1371/journal.pone.0151651)
Supplement: S3 Fig — X-axis: clustering threshold; Y-axis: number of clusters with 2x (red) or 3x (green line) coverage. The top panel shows within-sample, whereas the bottom panel shows among-sample clustering results. The optimal threshold optimizes the number of the clusters with 2x and 3x coverage. (DOCX) [file pone.0151651.s003.docx]

# Supporting information


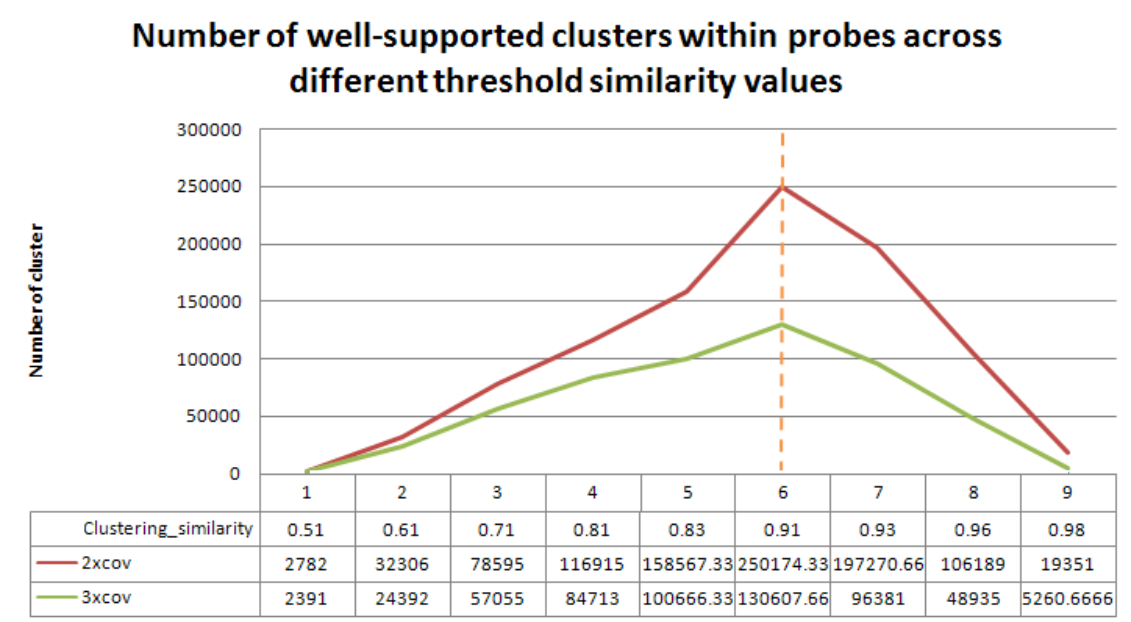


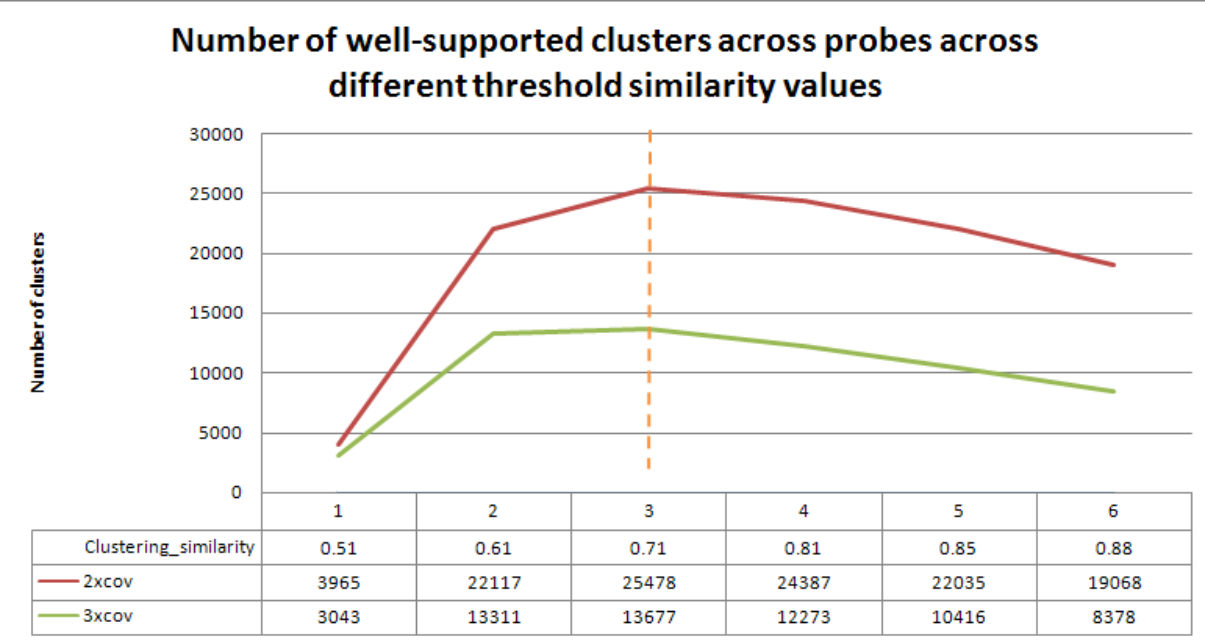


S3 Fig. Illustration of the clustering optimization of RAD-ref assembly clustering thresholds using Vsearch. *X*-axis: clustering threshold; *Y*-axis: number of clusters with 2x (red) or 3x (green line) coverage. The top panel shows within-sample, whereas the bottom panel shows among-sample clustering results. The optimal threshold optimizes the number of the clusters with 2x and 3x coverage.
