# Supplementary material for: Hybridization Capture Using RAD Probes (hyRAD), a New Tool for Performing Genomic Analyses on Collection Specimens
Source: PLoS One. 2016 Mar 21;11(3):e0151651. doi: 10.1371/journal.pone.0151651 (PMC4801390; doi:10.1371/journal.pone.0151651)

S4 Fig. Proportion of type II transitions to all the transitions and transversions for each of the reference catalog, with and without post-mortem bias correction. The data shown are for the fresh sample with DNA sonication and the museum samples without sonication. The left plot shows values without and the right plot with mapDamage2.0 correction.


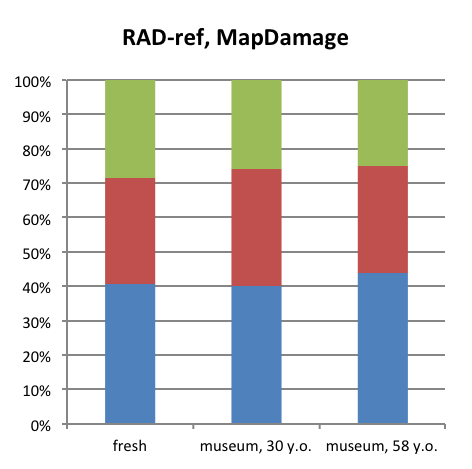

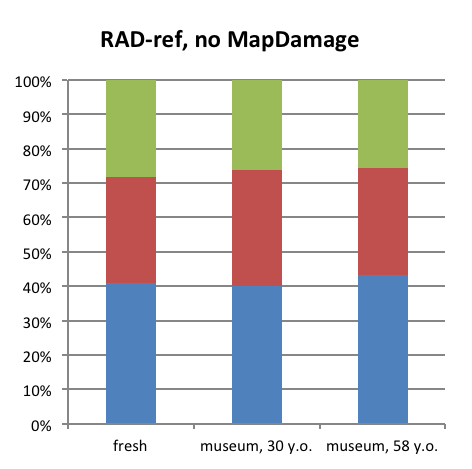

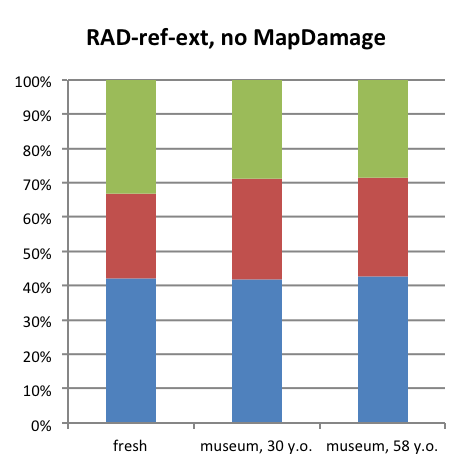

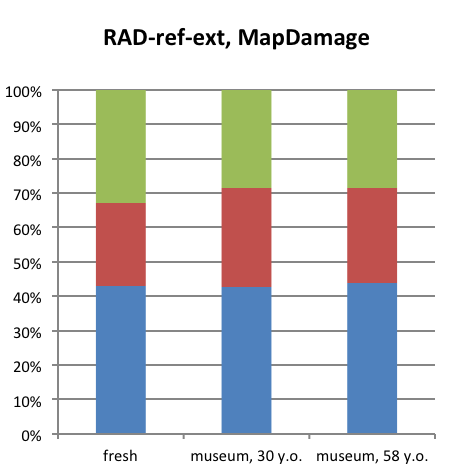

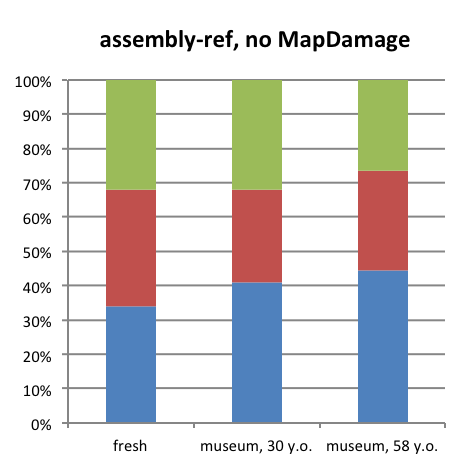

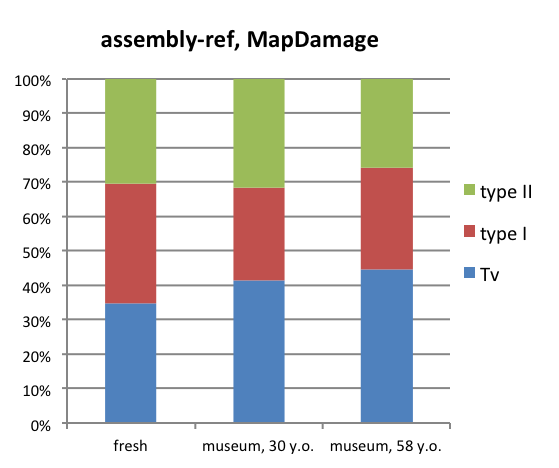

Supplement: S4 Fig — The data shown are for the fresh sample with DNA sonication and the museum samples without sonication. The left plot shows values without and the right plot with mapDamage2.0 correction. (DOCX) [file pone.0151651.s004.docx]
