# Supplementary material for: Hybridization Capture Using RAD Probes (hyRAD), a New Tool for Performing Genomic Analyses on Collection Specimens
Source: PLoS One. 2016 Mar 21;11(3):e0151651. doi: 10.1371/journal.pone.0151651 (PMC4801390; doi:10.1371/journal.pone.0151651)
Supplement: S1 Table — (DOCX) [file pone.0151651.s005.docx]

# Supporting information

S1 Table. mapDamage2.0 results based on each of the three reference catalogs for L. helle analyses, with the number of obtained SNPs (with and without application of mapDamage2.0).

| Sample type | | RAD-ref | | RAD-ext-ref | | assembly-ref | |
| --- | --- | --- | --- | --- | --- | --- | --- |
|  |  | no mapDamage | mapDamage | no mapDamage | mapDamage | no mapDamage | mapDamage |
| sonicated | fresh | 717 | 704 | 527 | 513 | 300 | 287 |
|  | museum, 30 y.o. | 1389 | 1366 | 956 | 939 | 842 | 825 |
|  | museum, 58 y.o. | 1284 | 1265 | 891 | 876 | 818 | 803 |
| non-sonicated | fresh | 198 | 196 | 187 | 183 | 109 | 109 |
|  | museum, 30 y.o. | 1348 | 1330 | 999 | 977 | 935 | 932 |
|  | museum, 58 y.o. | 1123 | 1110 | 889 | 875 | 866 | 864 |
